# Effect of timing of artificial insemination in relation to onset of standing estrus on pregnancy per AI in Nili-Ravi buffalo

**DOI:** 10.21451/1984-3143-AR2017-0015

**Published:** 2018-12-05

**Authors:** Umair Riaz, Mubbashar Hassan, Ali Husnain, Muhammad Ilyas Naveed, Jaswant Singh, Nasim Ahmad

**Affiliations:** 1 Department of Theriogenology, Faculty of Veterinary Science, University of Veterinary and Animal Sciences, Lahore, Pakistan.; 2 Western College of Veterinary Medicine, University of Saskatchewan, Saskatoon, SK, Canada.

**Keywords:** Nili-Ravi buffalo, timing of insemination, standing heat, pregnancy per AI

## Abstract

The aim of the present study was to determine the optimum time of artificial insemination after the beginning of standing estrus in buffalo. Nili-Ravi buffalo (n = 109) during breeding season were exposed to teaser bull at 12 hours interval to determine the standing heat (0 h). Buffalo were randomly allocated to different time groups and a single artificial insemination was performed either at 0 h (n = 30), 12 h (n = 27), 24 h (n = 28) or 36 h (n = 24). In a subset of buffalo (n = 38) ultrasonography was performed, twice daily from 0 h (onset of standing heat) to determine the time of ovulation. Pregnancy diagnosis was performed 35-40 days after AI. Results revealed that mean time of ovulation from onset of standing heat was 34.7 ± 0.96 h (range 30 to 42 h). Higher (P < 0.05) pregnancy per AI were achieved in buffalo when inseminated at 24 h (15/28; 53%) compared to 0 h (8/30; 26%) and 36 h (3/24; 13%). Pregnancy per AI, was in-between, in buffalo, inseminated at 12 h (10/27; 37%) and did not differ (P > 0.05) with those bred either at 24 h or 0 h. The odds ratio further confirmed that the occurrence of pregnancy per AI was two times higher in buffalo inseminated at 24 h as compared to those at 12 h. It is concluded that optimal pregnancy per AI can be achieved when buffalo are bred artificially 24 h after the onset of standing heat.

## Introduction

Artificial insemination (AI) has played a major role in genetic selection, disease control and cost effectiveness of breeding in dairy cattle ( Vishwanath, 2003 ; López-Gatius, 2012 ). Although, considerable research and development has taken place in estrus and ovulation synchronization in cows and buffalo with use of fixed time AI (Warriach *et al* ., 2007; Rossi *et al*., 2014 ; Yousuf *et al*., 2015 ), yet its application in vast majority of buffalo population of Asia is negligible. In contrast, traditional use of AI at detected estrus is much higher, however, fertility is generally compromised. Several factors which can affect fertility include heat stress ( Shenhe *et al*., 2018 ) semen quality, estrus signs, technician and timing of insemination in buffalo ( Anzar *et al*., 2003 ).

When AI was introduced in cattle in 1930’s ( Foote, 2002 ), the proper time of insemination became the most logical question. Series of classical experiments on ovulation and insemination timings addressed this issue in dairy cattle. Minimum number of cows returned to estrus when bred artificially 12 hours after the beginning of standing estrus ( Trimberger, 1948 ) that laid the foundation for AM-PM rule, i.e., a cow detected in standing heat in the morning should be bred artificially in the evening and vice versa. This method ensures sufficient time for sperm to capacitate and reach the proper site of fertilization in the oviduct ( Yanagimachi, 1981 ; Bedford, 1983 ). Owing to the anatomical and physiological similarities, a natural consequence was the adoption of AI methods in water buffalo from cattle protocols. Consequently, without gaining basic knowledge of timing of ovulation, AM-PM rule was adopted in the buffalo ( Drost, 2007 ). This practice resulted in reduced number of pregnancies leading to less adaptability of AI by the farmer community ( Anzar *et al*., 2003 ). Paradoxically, whether or not, this AM-PM rule holds true in water buffalo has not yet been systemically investigated.

Suboptimal pregnancy rates after AI could be a reason that genetic selection in buffalo did not take place at the same pace as in cows. AI must take place at a precise interval from ovulation thus ensuring successful fertilization. In case of early AI, the sperm would lose fertilizing ability ( Hawk, 1987 ) while in case of late AI, the ova would become aged which may cause failure of fertilization ( Hunter and Greve, 1997 ). With the expansion of AI programs in buffalo it was envisioned that the technique used in cattle required modifications in order to achieve the best results in buffalo ( Cockrill, 1974 ). Pioneer work in buffalo, based on rectal palpation, revealed that ovulation occurred at 34 h after the beginning of estrus ( Kanai and Shimizu, 1983 ), in contrast to 24 h in dairy cows after onset of standing heat ( Rajamahendran *et al*., 1989 ). Using ultrasonography, we reported that time of ovulation in buffalo was 31 h after the onset of standing heat ( Warriach *et al*., 2008 ). Higher conceptions were reported when AI was performed at later stage of estrus in buffalo under field conditions but without precise confirmation of ovulation ( Srivastava *et al*., 1998 ).

The reported delayed time of ovulation and lowered fertility in buffalo compared with dairy cows prompted us to hypothesize that a time interval of 24 h between onset of standing estrus and AI will be optimal to improve fertility in buffalo. There is no systematic study available which have shown the relationship of varying time of insemination with fertility. The objective of the present study was to determine the effect of insemination timing on pregnancy per AI in buffalo. The experimental design also allowed us to examine the optimal time interval between AI and ovulation time.

## Materials and methods

### Animals management

The present study was carried out at Livestock Experimental Station of Buffalo Research Institute, Pattoki, District Kasur, Punjab, Pakistan, during the months of September-December of 2012. Nili-Ravi buffalo (n = 109), multiparous, suckled and lactating, 4-6 years of age and 350-450 kg of body weight were maintained as untied under semi covered having free access to water, fed on 30–40 kg of seasonal fodder and 1–2 kg of vanda comprising of 15% crude protein and 65% total digestible nutrients to each buffalo daily. Buffalo were kept under optimal sanitary conditions. They were vaccinated against brucellosis, leptospirosis, clostridium, and IBR. The routine screening has been carried out and buffalo without any clinical and reproductive abnormalities were enrolled in this study. For the genital tract evaluation, buffalo were screened by ultrasonography (Honda; Model: HS-1500 Tokyo, Japan; 7.5 MHz). All buffalo included in the experiment came into estrus spontaneously.

### Estrus detection and time of insemination

Detection of estrus was performed two times in the day (6:00 a.m. and 6:00 p.m.) for duration of 30 minutes, with a penile deviated buffalo bull. Standing heat was considered in a buffalo when it did not move away for 5-7 seconds with the teaser bull being mounted on her. The buffalo were allotted randomly to be bred artificially either at 0 (n = 30), 12 (n = 27), 24 (n = 28) or 36 (n = 24) hours after the beginning of standing heat. Frozen thawed semen from a bull from Semen Production Unit, Qadirabad, Sahiwal with known fertility was used. One qualified technician performed the inseminations.

### Ultrasonographic examination and pregnancy diagnosis

Transrectal ultrasonography was used to measure the ovulation time with twelve hours interval, in a sub group of buffalo (n=38), which represented each group of timing of insemination after onset of standing heat, starting from the beginning of standing heat till ovulation. Ovulation was declared when the large dominant ovarian follicle present in the previous scan missing in the next one ( Pierson and Ginther, 1988 ). Time of ovulation was taken as the interval from beginning of standing estrus to mean of previous two scans (before ovulation and after ovulation). In three buffaloes ovulation did not occur till 48 h after the onset of standing heat were excluded from the data.

Transrectal ultrasonography at 35-40 days after artificial insemination was used for pregnancy diagnosis in this study. Positive pregnancy status was considered after observing the amniotic membrane, amniotic fluid and heartbeat of the embryo. Pregnancy per AI was determined as: [(number of buffalo pregnant divided by number inseminated) X 100].

### Statistical analysis

Differences in prediction of chances of pregnancy at different time intervals of AI, either at 0, 12, 24 or 36 hours after the onset of standing estrus was tested using binary logistic regression model. The significance of this model was determined using Wald Chi-square statistic, if it was statistically significant (i.e. P ≤ 0.05), odd ratios (OR) were used to predict the difference in likelihood of pregnancy between the AI groups. The OR were considered as, if OR = 1: no effect on pregnancy, OR > 1: higher incidence of pregnancy, OR < 1: lower incidence of pregnancy. Tests were assumed to be significant at P ≤ 0.05. Pregnancy per AI were analyzed using a Statistical Analysis System for Windows (SAS 9.2 Institute Inc., Cary, NC, USA).

## Results

### Effect of timing of artificial insemination on pregnancy

**Figure1:**
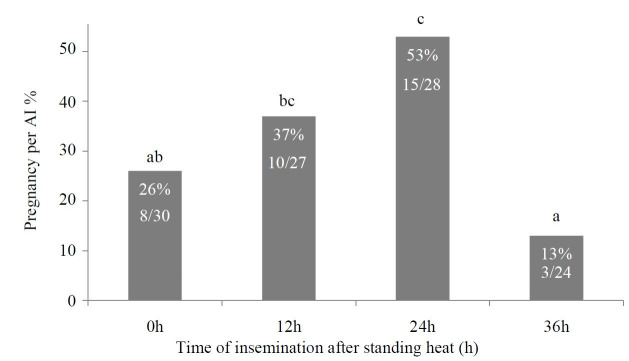
The effect of timing of AI on pregnancy per AI in buffalo inseminated on spontaneous estrus at different time intervals after the onset of standing heat. Different letters at each bar differ significantly (P< 0.05).

Timing of insemination with respect to beginning of standing estrus on pregnancy per AI in buffalo is presented in the Table 1 and Figure 1 . Maximum pregnancy per AI, 53% (15/28), was achieved in buffalo inseminated at 24 h after the onset of standing heat. This was followed by 37% (10/27) in those buffalo which were inseminated at 12 h after the beginning of standing estrus. Although, numerically there was a sixteen percent point difference between these two groups yet it was statistically non-significant. About 26% (8/30) buffalo became pregnant when they were inseminated just at the onset of standing heat (i.e. 0 h). This pregnancy per AI was significantly (P < 0.05) lower than those obtained at 24 h group but not (P > 0.05) from 12 h group. Fewer buffalo, became pregnant i.e., only 13% (3/24) when inseminated at 36 hours after the beginning of standing heat. Moreover, pregnancy per AI did not differ (P > 0.05) between buffalo inseminated at 0 and 12 (26% vs 37%) or 0 and 36 h groups (26% vs 13%). Comparison of pregnancy per AI between various groups using odds ratio revealed that its occurrence was almost 2 times more in buffalo inseminated at 24 h as compared to those inseminated at 12 h. Whereas, likelihood of buffalo to become pregnant were 3 and 8 times higher at 24 h compared with either 0 or 36 h after the beginning of standing estrus, respectively.

**Table 1:** The effect of timing of AI on pregnancy per AI after binary logistic regression analysis in buffalo inseminated on spontaneous estrus at different time intervals after the onset of standing heat.

AI Groups	Pregnancy per AI (%)	Odds ratio	P value
0 vs. 12 h	26 vs. 37	0.6	0.401
0 vs. 36 h	26 vs. 13	2.5	0.208
12 vs. 36 h	37 vs. 13	4.1	0.054
24 vs. 0 h	53 vs. 26	3.1	0.039
24 vs. 12 h	53 vs. 37	1.9	0.220
24 vs. 36 h	53 vs. 13	8.0	0.003

P ≤ 0.05 shows the significant differences between AI groups. Odd ratios (OR) shows the occurrence to become pregnant in AI groups, if OR = 1: no effect on pregnancy, OR > 1: increased occurrence of pregnancy, OR < 1: decreased occurrence of pregnancy.

### Follicular characteristics, timing of ovulation

The average timing of ovulation in buffalo was 34.5 ± 0.96 h (range: 30 to 42 h) from the beginning of standing estrus. Average growth rate of the ovulatory follicle was 1.9 ± 0.06 mm/day from the beginning of standing estrus to ovulation. Average size of ovulatory follicle just before the ovulation was 14.09 ± 0.24 mm.

## Discussion

This study to the best of our knowledge is first and prospective, reporting most appropriate time for artificial breeding in relation to the beginning of standing estrus. The outcomes of the present experiment are new and directly applicable in the field. The pregnancy per AI was highest (53%) when buffalo of the present study were bred artificially at 24 h, moderate (37%) at 12 h, and lower (23%) either at 0 h or (13%) at 36 h, after the beginning of standing estrus. Similarly, higher pregnancy rate (50%) was observed in smaller number of buffalo in field conditions when they were inseminated late i.e., around 23-26 h after the beginning of heat signs ( Srivastava *et al*., 1998 ). Pregnancy rate in buffalo using frozen semen was low (37-41%) with single insemination ( Andrabi, 2009 ). In order to circumvent this, double inseminations, with 12 hour interval, after initiation of estrus, were adopted in buffalo ( Neglia *et al*., 2003 ; Naseer *et al*., 2011 ). Pregnancy rate were reported to be fairly high (upto 60%) in synchronized and (upto 80%) in resynchronized buffalo with fix time inseminations (de Araujo Berber *et al* ., 2001; Baruselli *et al*., 2001 ; Warriach *et al*., 2008 ; Salzano *et al*., 2017 ; Arshad *et al*., 2017 ). The higher pregnancy rate, achieved at 24 hours with single AI in this study, is perhaps due to artificial insemination time with regard to timing of ovulation. The major strength of our study is that it is controlled and precise; however, smaller number of buffaloes used may be considered as a caveat. Clearly, this type of prospective study is not easy to carry out from farm managerial point of view as no farmer would allow inseminations of buffalo at odd timings. Collectively, these data imply that breeding management strategies need modification in buffalo. Although the pregnancy per AI did not differ significantly in buffalo inseminated either at 12 h and 24 h after the beginning of standing heat using Wald Chi-square. However, the odds ratio revealed that the occurrence of pregnancy per AI was two times higher in buffalo inseminated at 24 h as compared to those at 12 h. It would be interesting to confirm these findings in other breeds of buffalo on larger sample size to check differences in fertility between the 12 (standard AM-PM, PM-AM rule) and 24h (AM-AM and PM-PM) intervals.

Timing of ovulation is intimately associated with proper time of insemination for the success of AI ( Nalbandov and Casida, 1942 ). This study revealed that ovulation occurred about 35 h after the beginning of standing estrus in buffalo. A study by ( Moioli *et al*., 1998 ) reported this time to be about 53 h after initiation of continuous courtship in buffalo. In contrast, time of ovulation was 24 hours after the beginning of standing estrus in dairy cows as observed by Rajamahendran *et al*. (1989) . These data clearly demonstrate that the interval from onset of standing heat-ovulation is significantly (at least about eight hours) more in buffalo than cow. This late occurrence of ovulation would, therefore, require delayed insemination in buffalo, compared to cow, to acquire better results of AI.

The findings of the present work provide some physiological insight of the mechanism of fertilization in buffalo. Lower pregnancy per AI in buffalo when inseminated at 0 and 36 h after onset of standing heat, are most likely due to mismatching of the sperm and ova at the site of fertilization. At early insemination (0 h) sufficient viable sperm might still not be available for fertilization. In case of much delayed insemination (36 h), the sperms have to undergo capacitation first which takes almost 8 hours ( Hunter and Wilmut, 1983 ) and perhaps by that time ova after ovulation might have become aged. Data from studies on polytocous species, like rabbits ( Tesh, 1969 ) and pigs ( Soede *et al*., 1995 ), have reported a decrease in the number of fertilized oocytes when AI was done earlier (above than 29 and 32 h earlier to ovulation, respectively). The experimental confirmation in buffalo about the life span of spermatozoa and of ovum after ovulation in female genital tract is not still available.

The novelty of the present studies lies in the proposal of reframing the timing of insemination in buffalo, for optimum fertility, that differs from the cow. The AM-PM rule i.e. interval between onset of standing estrus and AI, which was classically developed in dairy cows in United States during late forties, was implemented, without any systematic experimental study, in buffalo. Resultantly, the fertility remained generally low in buffalo ( Andrabi *et al*., 2001 ). With the evidence of delayed time of ovulation in buffalo as compared to cows and higher fertility (53%) with late insemination (24 h) in this study, the AM-PM rule appears to be misfit for buffalo and need to be modified as AM-AM or PM-PM.

## Conclusion

The maximum pregnancy per AI was obtained when buffalo were inseminated 24 hours after the beginning of standing estrus. The time of ovulation is about 35 h after the beginning of standing estrus and is clearly late compared to cows. This implies that the breeding management needs to be modified in buffalo.
